# Supplementary material for: Epigenetic control of tetrapyrrole biosynthesis by m4C DNA methylation in a cyanobacterium
Source: DNA Res. 2024 Dec 7;31(6):dsae035. doi: 10.1093/dnares/dsae035 (PMC11662147; doi:10.1093/dnares/dsae035)
Supplement: dsae035_suppl_Supplementary_Tables_S1-S6_Figures_S1-S9 [file dsae035_suppl_supplementary_tables_s1-s6_figures_s1-s9.pdf]

Epigenetic control of tetrapyrrole biosynthesis by ^{m4}C DNA methylation in a cyanobacterium

Nils Schmidt[1], Nils Stappert[2], Kaori Nimura-Matsune[3], Satoru Watanabe[3], Roman Sobotka[4,5], Martin Hagemann[1]*, Wolfgang R. Hess[2*]

[1]Institute of Biosciences, Department of Plant Physiology, University of Rostock, D-18059 Rostock, Germany

[2]University of Freiburg, Faculty of Biology, Genetics and Experimental Bioinformatics, Schänzlestr. 1, D-79104 Freiburg, Germany

[3]Department of Bioscience, Tokyo University of Agriculture, Tokyo, Japan.

[4]Institute of Microbiology of the Czech Academy of Sciences, Opatovický mlýn, Třeboň, 379 01, Czech Republic

[5]Faculty of Science, University of South Bohemia, České Budějovice, 370 05, Czech Republic

***Corresponding authors:** Martin Hagemann, University of Rostock, Institute of Biosciences, Department of Plant Physiology, A.-Einstein-Str. 3, D-18059 Rostock, Germany; Tel: +49(0)3814986110; Email: martin.hagemann@uni-rostock.de

Wolfgang R. Hess, University of Freiburg, Faculty of Biology, Genetics and Experimental Bioinformatics, Schänzlestr. 1, D-79104 Freiburg, Germany; Tel: +49(0)7612032796; Email: wolfgang.hess@biologie.uni-freiburg.de

Supplementary Material

Supplementary Datasets: **p. 2**

Supplementary Tables: **p. 3**

Supplementary Figures: **p. 8**

Supplementary References: **p. 17**

Supplementary Datasets

Supplementary Dataset S1. Details of qRT-PCR analysis and statistical test in Figure 2A. Excel sheet carrying detailed information about the qRT-PCR analyses, including Cq values, Cq SD, Cq threshold, baseline, RQ values for each sample. Negative control (NTC) is a reaction where the reverse transcriptase was replaced by H_2O. Sample describes the strains examined where numbers correspond to the biological replicates. Targets are the examined genes of interest. Reporter is the dye used for the reaction. The Excel sheet is separated into different tabs carrying information about overall results, statistical calculations, run information, sample setup, amplification data, melt curve raw data and melt curve results.

See separate Excel file.

Supplementary Dataset S2. Details of qRT-PCR analysis and statistical test in Figure 2B. Excel sheet carrying detailed information including Cq values, Cq SD, Cq threshold, baseline, etc., for each sample. Negative control (NTC) is a reaction where the reverse transcriptase was replaced by H_2O. Sample describes the strains examined where numbers correspond to the biological replicates. Targets are the examined genes of interest. Reporter is the dye used for the reaction. The Excel sheet is separated into different tabs carrying information about overall results, statistical calculations, run information, sample setup, amplification data, melt curve raw data and melt curve results.

See separate Excel file.

Supplementary Dataset S3. Location of all mutations found in the resequencing analysis of Δ*sll0729* and pseudorevertant strains compared with the parental WT strain. Seven pseudorevertant strains (Sup1 to Sup7) were sequenced together with their parent strains (KO1 and KO2) and the used wild-type strain of *Synechocystis* 6803.

See separate Excel file.

Supplementary Tables

Supplementary Table S1. Deoxynucleotide primers used in the study. All sequences are given in 5’ to 3’ direction; reverse transcriptase (RT).

ID	Name	Sequence (5' to 3')	Description	Purpose
P1	RT-qPCR_slr1790-rv	ATAGACAAAAAGACGCAC	For RT reaction to synthesize cDNA of *slr1790* mRNA.	Quantitative RT PCR
P2	RT-qPCR_rnpA-rv	TTAGACGATTTCTAACCGT	For RT reaction to synthesize cDNA of *rnpA* mRNA.	
P3	RT-qPCR_luxAB-fw	TCTTCCTAACAGGTTAGC	For RT reaction to synthesize cDNA of *luxAB* mRNA.	
P4	qPCR_slr1790-fw	AAGCGCGAGTATTTCTCC	For qPCR amplification of *slr1790.*	
P5	qPCR_slr1790-rv	ATCCAGCAAACCAAACAACA		
P6	qPCR_rnpA-fw	CCAGACCGTTTATCAGCAAG	For qPCR amplification of *rnpA.*	
P7	qPCR_rnpA-rv	TTTTGGCTGACGGTGATG		
P8	qPCR_luxAB-fw	GCAGCAACAAATAAATTTCCCG	For qPCR amplification of *luxAB.*	
P9	qPCR_luxAB-rv	ATCGCTTTGTTCGGCTTG		
P10	slr1790-AQ-GGCC-rv	AAAAACAAATATTTCCAAACTTCATTTCCCAGGAACAGGGTGGGTC	To amplify promoter *slr1790* WT variant and use it for Aqua cloning into pILA vector directly upstream of *luxAB*.	Generation of luciferase strains carrying promoter variants of *slr1790* upstream of *luxAB*
P11	slr1790-AQ-GGTC-rv	AAAAACAAATATTTCCAAACTTCATTTCCCAGGAACAGGGTGGGTCATCAGCTCATACCCAAGGGGACCATTATATCGCCTTGC	To amplify promoter *slr1790* GGTC variant and use it for Aqua cloning into pILA vector directly upstream of *luxAB*.	
P12	slr1790-AQ-GGGC-rv	AAAAACAAATATTTCCAAACTTCATTTCCCAGGAACAGGGTGGGTCATCAGCTCATACCCAAGGGGCCCATTATATCGCCTTGC	To amplify promoter *slr1790* GGGC variant and use it for Aqua cloning into pILA vector directly upstream of *luxAB*.	
P13	slr1790-AQ-GGAC-rv	AAAAACAAATATTTCCAAACTTCATTTCCCAGGAACAGGGTGGGTCATCAGCTCATACCCAAGGGGTCCATTATATCGCCTTGC	To amplify promoter *slr1790* WT variant and use it for Aqua cloning into pILA vector directly upstream of *luxAB*.	
P14	slr1790-AQ-fw	GGGATCCAATTGGCAGTGCAGGTCGATACACTCCCTTGGTTTTCACCATTGCC	To amplify promoter *slr1790* and use it for Aqua cloning.	

P15	slr1790Mufw_190	ACGGTTTCTTCCGC TATGAC	To amplify promoter *slr1790* WT variant to clone into pJet1.2.	Generation of promoter variants of *slr1790* with a 5' inserted spectino-mycin resistance marker gene
P16	slr1790Murv_129 7	CAACAGTAACGCCA CAAAGG	To amplify promoter *slr1790* WT variant to clone into pJet1.2 and confirm segregation with 5’ upstream inserted *aadA.*	
P17	Promoter_Mut-P	GGACTGGGCAAGGC GATATAATGGTCCC CTTGGGTATGAGCT GATGACC	Mutagenesis primer to mutate GGCC to GGTC 4 nt upstream of the transcription start within the *slr1790* promoter.	
P18	Sm fw	ACGAACCCAGTGGA CATAAG	Verification of antibiotic resistance cassette *aadA.*	
P19	Sm rev	TCAGGAACCGGATC AAAGAG		
P20	slr1790Murv_250 2	AGCTTTCGAGTGCC ATTGAC	Sequencing of promoter *slr1790* GGCC/GGTC motif 4 nt upstream of the *slr1790* transcription start.	
P21	Pslr1790_121rev	GGACTTGGTTTAAC CCTCCAAATC	Primer to confirm segregation of promoter *slr1790* with 5’ upstream inserted *aadA.*	
P22	del0729:0729rv	GGGGAAATAAATAA ATCAGC	Primer to confirm segregation of *sll0729_aphII*.	Generation of *sll0729* knockout strains
P23	del0729:0729fw	AACTCTTTACCTTTG GAAGC		

Supplementary Table S2. Overview of genetically modified *Synechocystis* sp. PCC 6803 strains.

**Strain**	**Characteristics**	**Resistance**
P*slr1790*	Strain in which native *slr1790* promoter (GGCC motif intact) drives *hemJ* transcription; fused to antibiotic resistance cassette *aadA* inserted 237 bp upstream of *slr1790* transcription start; control to MP*slr1790*.	spectinomycin
MP*slr1790*	Mutated *slr1790* promoter (GGCC changed to GGTC) driving *hemJ* transcription and fused to antibiotic resistance cassette *aadA* 237 bp upstream of *slr1790* transcription start.	spectinomycin
Δ*sll0729*	Deletion of *sll0729* encoding M.Ssp6803II with inserted antibiotic resistance cassette *aphII* located in the chromosome at position 3.424.686 to 3.425.512 in sense orientation.	kanamycin
P*slr1790*/Δ*sll0729*	Native *slr1790* promoter (GGCC motif intact) driving *hemJ* transcription and fused to antibiotic resistance cassette *aadA* 237 bp upstream of *slr1790* transcription start in Δ*sll0729* background.	spectinomycin, kanamycin
MP*slr1790*/Δ*sll0729*	Mutated *slr1790* promoter (GGCC changed to GGTC) driving *hemJ* transcription and fused to antibiotic resistance cassette *aadA* 237 bp upstream of *slr1790* transcription start in Δ*sll0729* background.	spectinomycin, kanamycin
Suppr. Δ*sll0729*	deletion of *sll0729* encoding M.Ssp6803II with inserted antibiotic resistance cassette *aphII* in suppressor strain background	kanamycin
P*slr1790* ggCc *luxAB*	Chromosomal integration of *luxAB* with the native promoter *slr1790* in *luxCDE* background.	kanamycin, chloramphenicol
P*slr1790* ggTc *luxAB*	Chromosomal integration of *luxAB* with GGCC to GGTC mutated *slr1790* promoter in *luxCDE* background	kanamycin, chloramphenicol
P*slr1790* ggAc *luxAB*	Chromosomal integration of *luxAB* with GGCC to GGAC mutated *slr1790* promoter in *luxCDE* background.	kanamycin, chloramphenicol
P*slr1790* ggGc *luxAB*	Chromosomal integration of *luxAB* with GGCC to GGGC mutated *slr1790* promoter in *luxCDE* background.	kanamycin, chloramphenicol

Supplementary Table S3. Mutations mapped in the Δ*sll0729* suppressor mutants S1 to S7 (S, strain), compared with the parental Δ*sll0729* strain. The GenBank (Gbk) accession numbers of the respective replicon is given in the 2nd column, followed by the nucleotide positions (P), the type of mutation (T), with either single nucleotide variation (S) or deletion (D), followed by the respective nucleotides (Nt) and identity of the mutated allele (A), the mutation result (R; dis, promoter discriminator region; fs, frame shift; si, silent), the locus tag (L) of the associated gene, ORF ID (ORF) and gene product. Further details can be found in **Supplementary Dataset S3.**

S	Gbk	P	T	Nt	A	R	L	ORF	Product
S1, S2, S3, S4, S5, S6, S7	CP003265	256605	S	C	T	dis	MYO_ 12320	*slr1790*	protopor-phyrinogen IX oxidase, HemJ
S1	CP003265	487987 – 487991	D	TCCTC	-	fs	MYO_ 14540	*slr1609*	long-chain-fatty-acid CoA ligase
S1	CP003265	703647	D	A	-	fs	MYO_ 16420	*slr1393*	cyanobacterio-chrome
S3, S4	CP003265	1763737	S	A	G	Leu > Pro	MYO_ 116270	*sll1895*	EAL domain protein
S2	CP003265	2262055 – 2262059	D	ATGGT	-	5’ UTR	MYO_ 120720	*ssl1807*	membrane protein
S5, S6, S7	CP003265	2867061	S	G	A	si	MYO_ 126030	*slr0073*	sensory transduction histidine kinase

Supplementary Table S4. Standard deviation and significance of cell size differences. A total of 110 cells was measured for each strain. The *p*-values were calculated towards the wild-type values. This Table extends the data in **Supplementary Fig. S4.**

	Average area [μm²]	variance	standard deviation	*p*-value
**WT**	4.80	1.30	1.14	-
**Δ*sll0729***	3.91	1.11	1.05	6.18739E-09
**P*slr1790***	6.00	2.22	1.49	1.0087E-10
**MP*slr1790***	5.51	1.67	1.29	1.03697E-05
**P*slr1790*/Δ*sll0729***	3.52	0.83	0.91	5.4733E-17
**MP*slr1790*/Δ*sll0729***	3.02	0.42	0.65	6.60528E-31

Supplementary Table S5. Standard deviation and significance of PPIX values. The *p*-values were calculated towards the wild-type values. These data extend the results shown in **Table 1**.

	PPIX	variance	standard dev.	*p*-value
**WT**	49.2	2111.2	45.9	-
**Δ*sll0729***	3921.2	4635178.1	2152.9	0.017
**P*slr1790***	32.4	636.3	25.2	0.355
**MP*slr1790***	26.5	499.7	22.4	0.222
**P*slr1790*/Δ*sll0729***	3058.4	1865476.3	1365.8	3.52E-07
**MP*slr1790*/Δ*sll0729***	15.3	316.5	17.8	0.078

Supplementary Table S6. Standard deviation and significance of CoPP values. The *p*-values were calculated towards the wild-type values. These data extend the results shown in **Table 1**.

	CoPP	variance	standard dev.	*p*-value
**WT**	65.1	388.14	19.70	-
**Δ*sll0729***	269.7	71077.08	266.60	0.007
**P*slr1790***	51.5	637.81	25.26	0.085
**MP*slr1790***	56.6	1423.01	37.72	0.266
**P*slr1790*/Δ*sll0729***	430.8	74011.41	272.05	5.58061E-05
**MP*slr1790*/Δ*sll0729***	34.8	434.50	37.723	0.003

Supplementary Figures

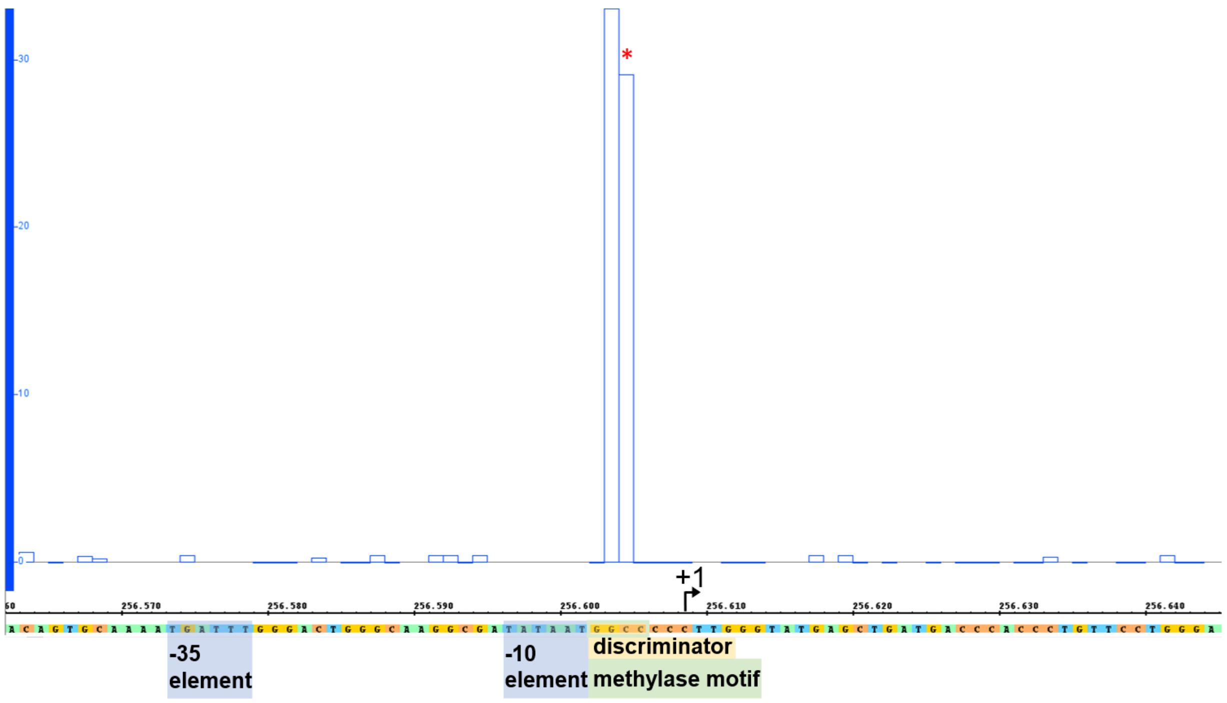

Supplementary Figure S1. The GGCC motif in the *slr1790* promoter is fully methylated in wild-type DNA. Bisulfite sequencing data revealed that the C at pos. 256,605 (marked by red asterisk) is methylated in wild-type cells. Transcriptional start site is indicated as a black arrow (+1). Previously generated bisulfite raw data are available at https://www.ncbi.nlm.nih.gov/biosample/8378604 (BioProject ID: PRJNA430784, BioSample: SAMN08378604, Run: SRX3574087). This figure extends **Fig. 1**.

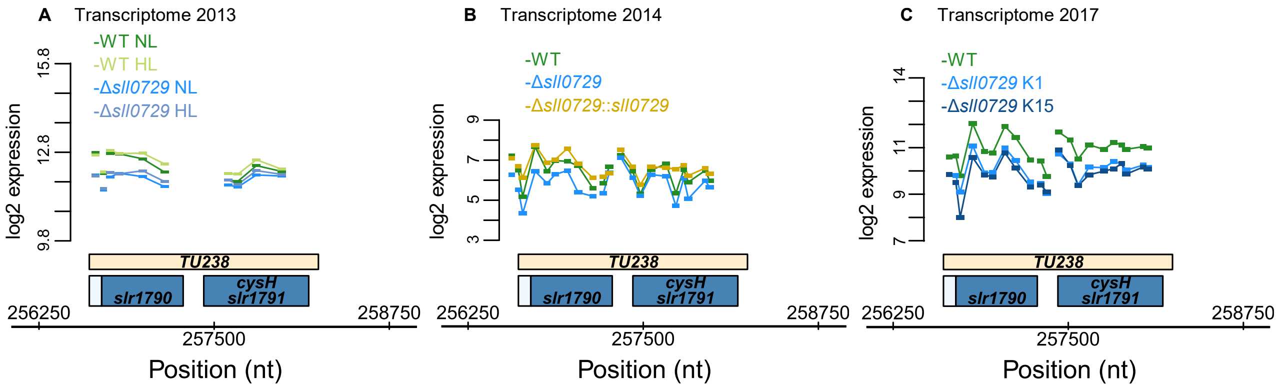

Supplementary Figure S2. The absence of M.Ssp6803II and connected GGCC methylation/unmethylation impacts *hemJ* expression. Visualization of the microarray results obtained for the *hemJ-cysH* (*slr1790-slr1791*) locus from three previous analyses with different cultivation schemes and microarray designs. In all analyses, samples from wild type (WT) were compared to an Δ*sll0729* mutant. The two genes are transcribed in a joint transcriptional unit, TU238.[1] **(A)** Experiment performed in 2013 that included a shift from standard light conditions (NL) to high light (HL). (**B)** Experiment performed in 2014 with a freshly made Δ*sll0729* mutant and a strain in which an intact *sll0729* allele was expressed for complementation (strain Δ*sll0729::sll0729*). Note that the array design differed in from that one used in panel (A). Results from this analysis were also used in the publication Gärtner et al. (2019).[2] (**C)** Experiment performed in 2017, same array design as in panel (B), but with two independently obtained new Δ*sll0729* deletion mutants (Δ*sll0729* K1 and K15). This figure extends **Fig. 2A**.

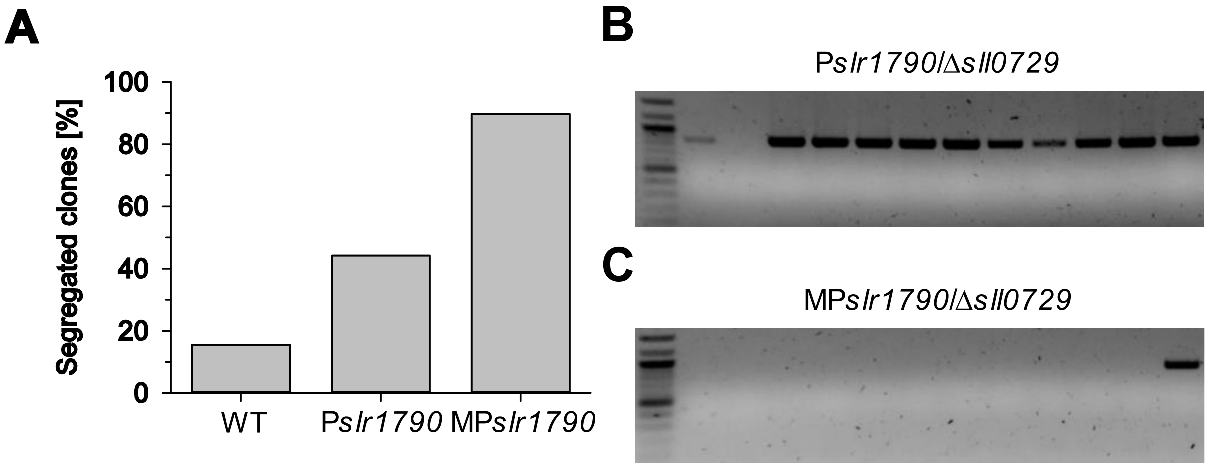

Supplementary Figure S3. Ratio of successful segregation of the *sll0729* mutation in different *Synechocystis* 6803 strains. The construct for the deletion of *sll0729* was transformed into cells of the *Synechocystis* 6803 wild type (WT) leading to mutant Δ*sll0729*, the strain with the native (P*slr1790*), and with the mutated *hemJ* promoter (MP*slr1790*). Kanamycin-resistant clones were isolated and the segregation status of the *sll0729* mutation was analyzed via PCR. (**A)** The percentage of completely segregated clones, i.e. where the gene *sll0729* was completely deleted, is displayed. (**B, C)** PCR showing a 827 bp fragment for non-segregated clones of P*slr1790*/Δ*sll0729* and MP*slr1790*/Δ*sll0729*. Only few clones of P*slr1790*/Δ*sll0729* were segregated; however, nearly all MP*slr1790*/Δ*sll0729* were segregated. This figure extends **Fig. 3A,B**.

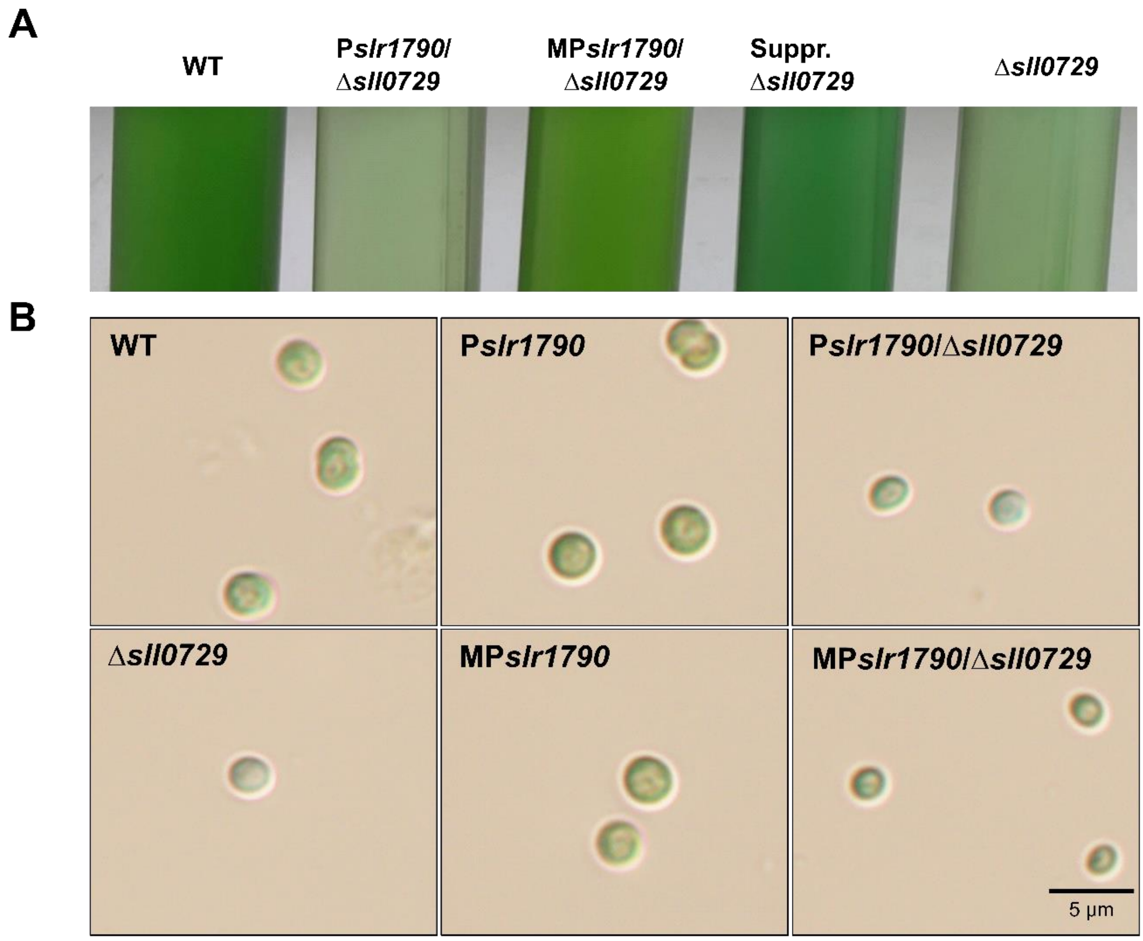

Supplementary Figure S4. Phenotypic alterations in different strains of *Synechocystis* 6803 with mutated *sll0729* gene and/or mutated *hemJ* promoter. (**A**) Phenotypes, i.e. optical appearance of the different strains. Note the bluish, less pigmented appearance of the mutant Δ*sll0729* and the Δ*sll0729* in the background of the native promoter (P*slr1790*), whereas the pigmentation became WT-like in the suppressor clone (Suppr. Δ*sll0729*) and the strain with mutated *sll0729* gene in the background of the mutated *hemJ* promoter (MP*slr1790*). See **Supplementary Table S4** for numerical values and statistical analysis. (**B**) Microscopy images from strain with deleted Δ*sll0729* and *hemJ* promoter mutants. The images where taken at 100x magnification. Please note the different pigmentation of single cells. This figure extends **Fig. 3C,D**.

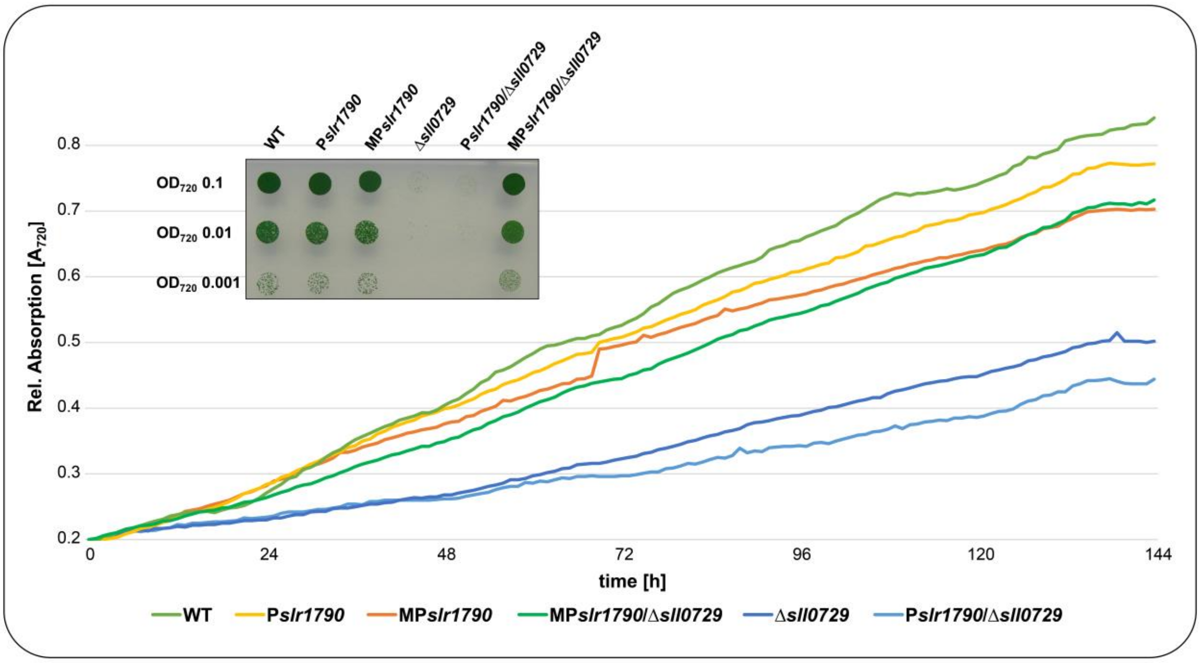

Supplementary Figure S5. Growth of different strains of *Synechocystis* 6803 with mutated *sll0729* gene and/or mutated *hemJ* promoter on solid (inset) or in liquid medium. The strain details can be found in **Supplementary Table S2**. This figure extends **Fig. 3C,D**.

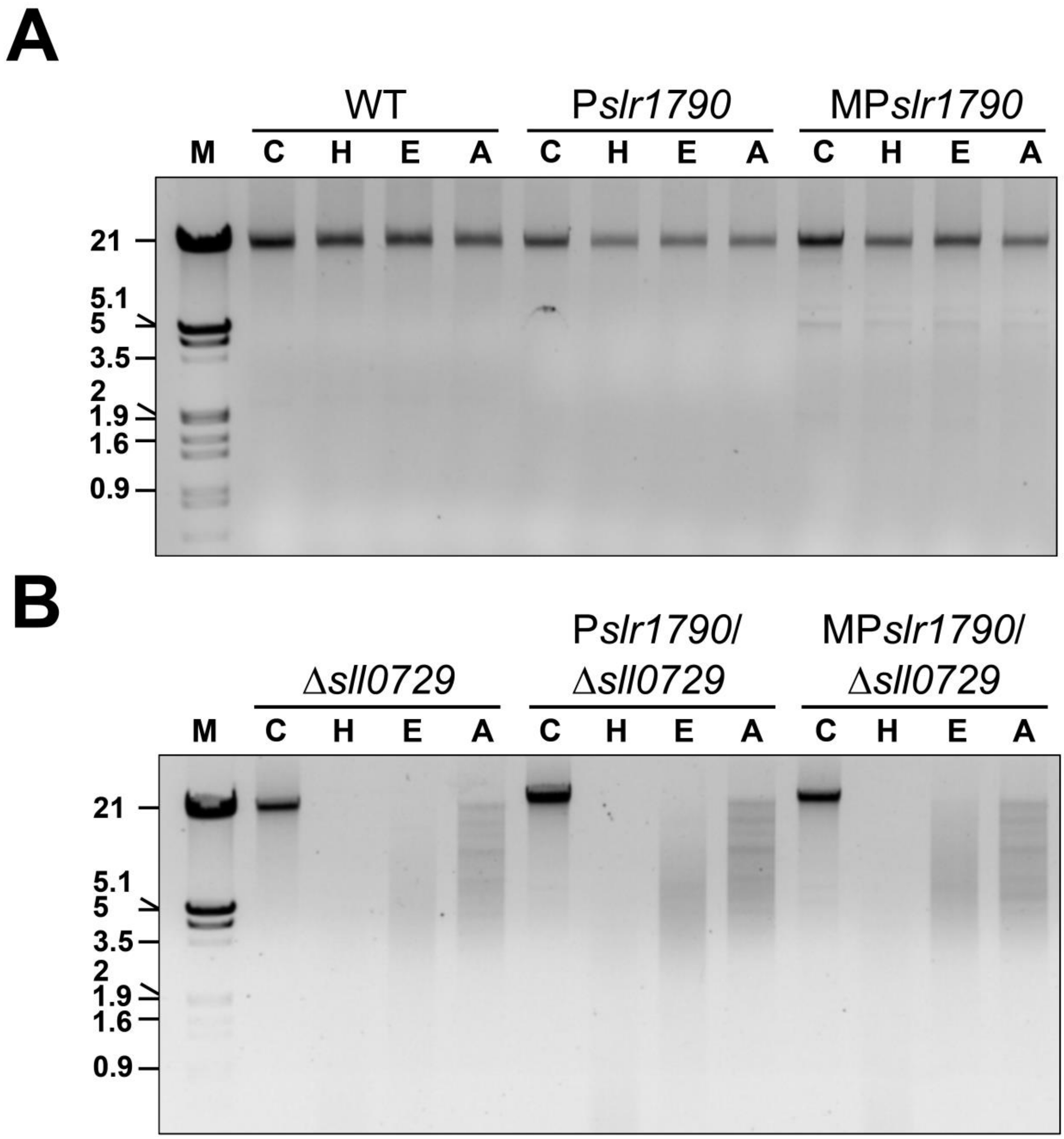

Supplementary Figure S6. Assay for GGCC methylation of different *Synechocystis* 6803 strains by restriction analysis. (A) Strains with intact *sll0729* gene expressing M.Ssp6803II. (**B)** Strains with deleted *sll0729* gene not expressing M.Ssp6803II. Three µg total DNA from each strain was treated by *Hae*III (GG/CC), *Eae*I (Y/GGCCR), or *Apa*I (GGGCC/C) for 16 h at 37°C.

These three restriction enzymes bear GGCC motifs in their recognition sequences but cannot cut DNA with methylated $GG^{m4}CC$ sites. (M = Marker, combination of bacteriophage λ DNA cut with *Eco*RI and separately with *Hind*III; C = uncut control; H = *Hae*III; E = *Eae*I; A = *Apa*I).

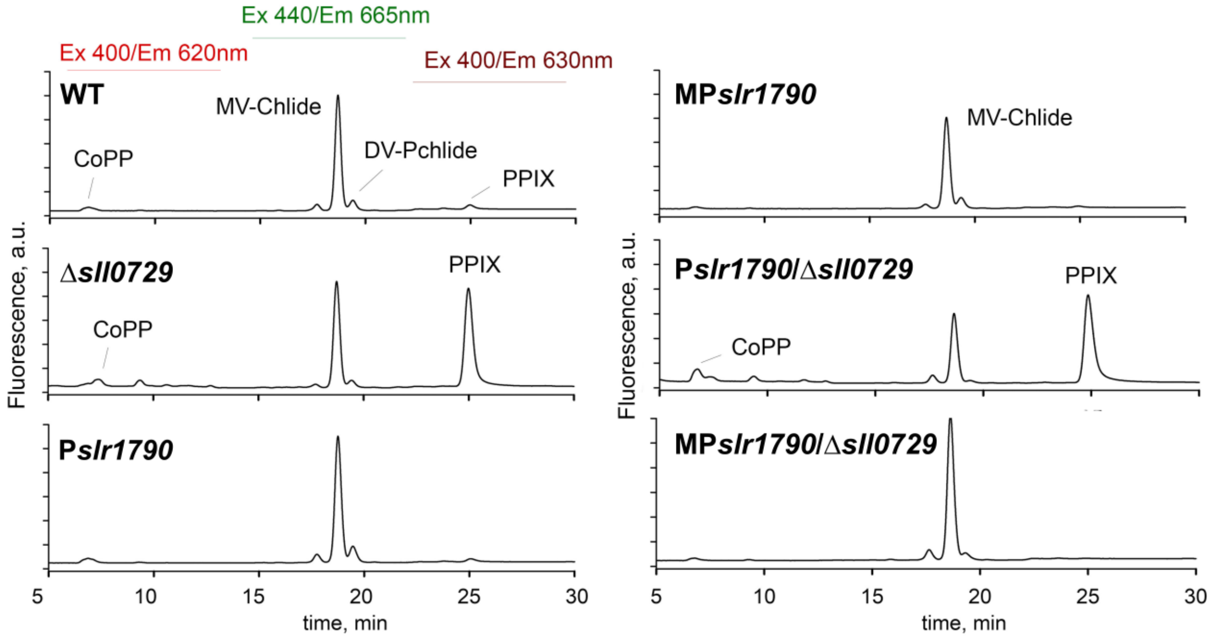

Supplementary Figure S7. HPLC analysis of *Synechocystis* PCC 6803 pigments. Chromatograms were recorded by fluorescent detector (Agilent 1260); settings of excitation and emission wavelengths during analysis is indicated. CoPP = coproporphyrin III; DV-Pchlide = divinyl-protochlorophyllide; MV-Chlide = monovinyl-chlorophyllide; PPIX - protoporphyrin IX. This figure extends **Table 1**.

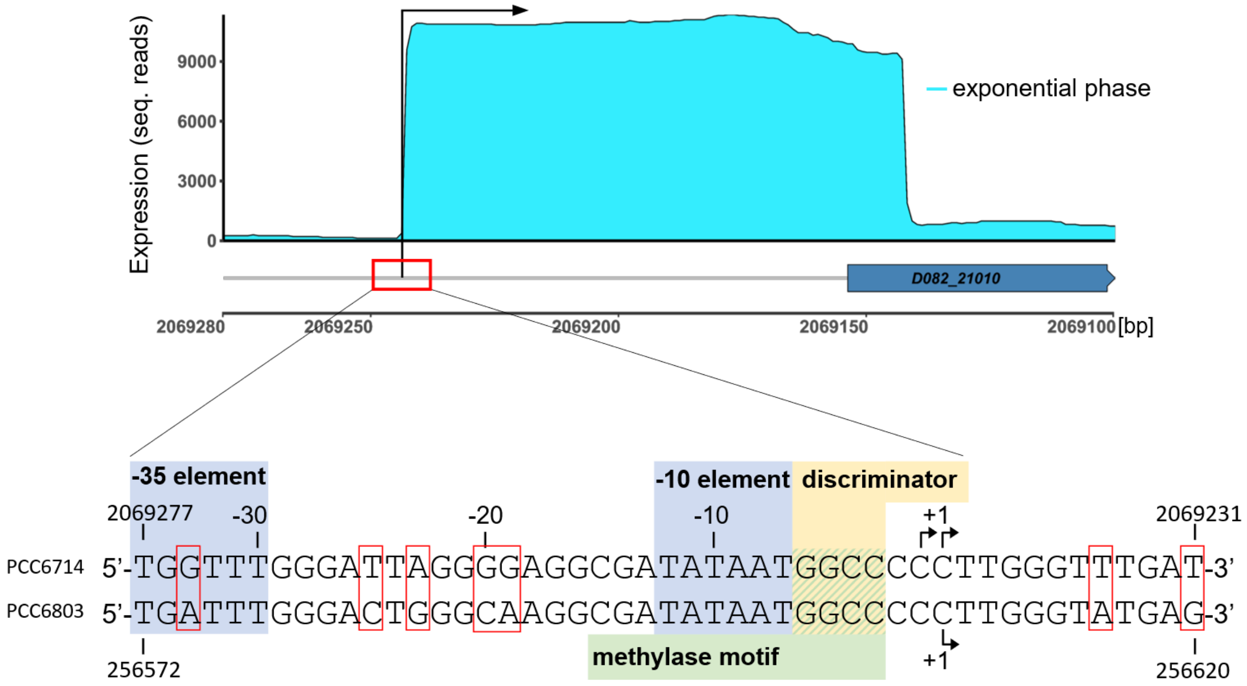

Supplementary Figure S8. The regulation of *hemJ* via GGCC-specific methylation might be conserved among cyanobacteria. In the related strain *Synechocystis* 6714 a GGCC-methylation motif is situated between the -10 promoter element and the transcription start site of gene D082-21010 that encodes the HemJ protein (upper sequence).

For comparison, the *Synechocystis* 6803 *slr1790* promoter sequence is included (lower sequence). Sequence differences are boxed in red. This figure extends **Fig. 1**.

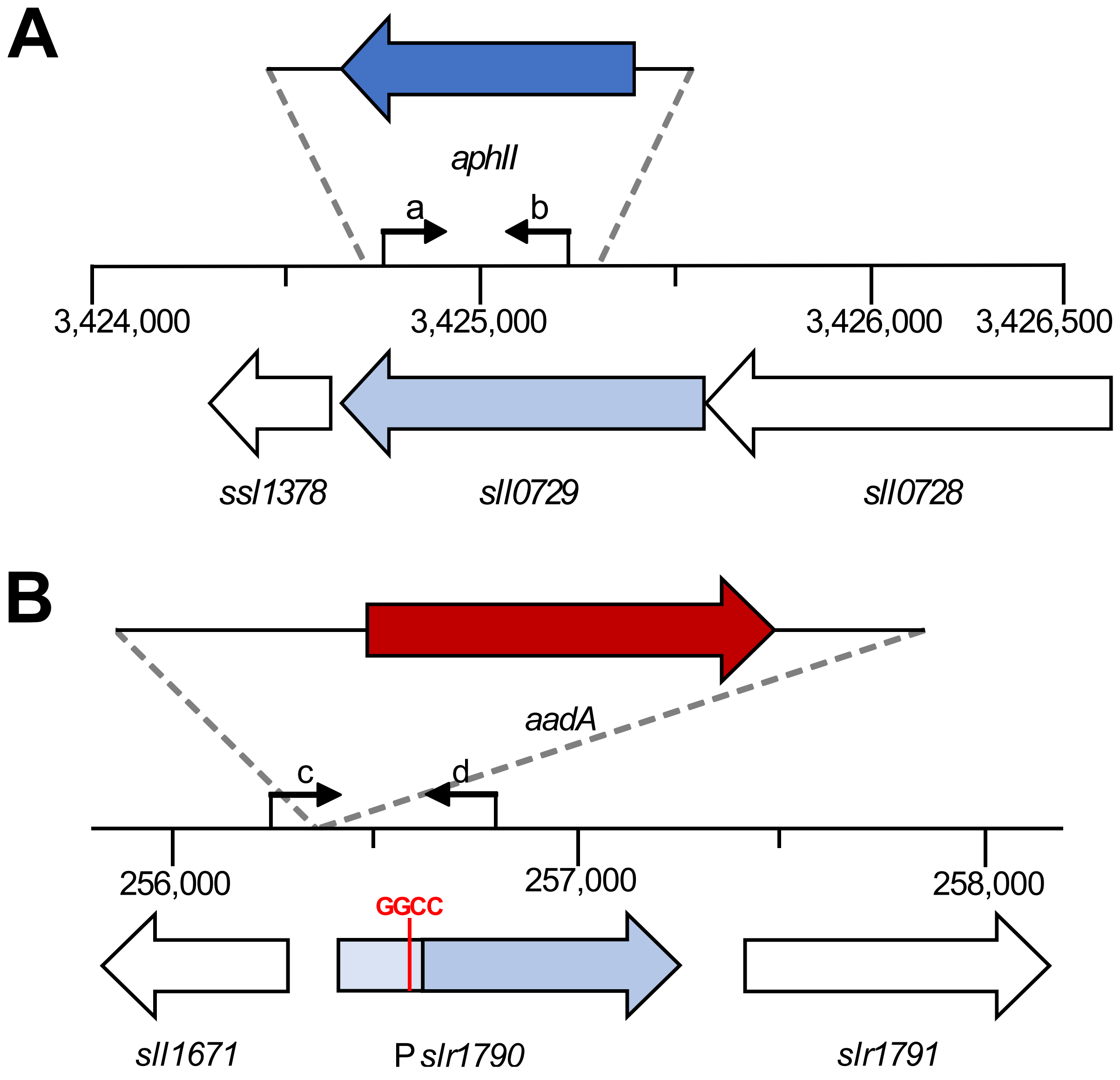

Supplementary Figure S9. Schematic view of P*slr1790* and Δ*sll0729* mutant. (A) The primers to confirm the segregation (P22 and P23) are binding at positions **a** and **b**, resulting in an 827 bp fragment in WT and no fragment in all Δ*sll0729* strains. This figure supports **Supplementary Fig. S3B,C**. **(B)** Segregation primers (P15 and 21) bind in all the P*slr1790* and MP*slr1790* strains at positions **c** and **d**, yielding a 639 bp PCR-fragment in WT and a 2,687 bp fragment in all P*slr1790* and MP*slr1790* strains. The *slr1790* 5' UTR and promoter region is boxed in light blue. This figure supports **Fig. 3A**. The numbers correspond to the genomic positions.

Supplementary References

1. Kopf, M., Klähn, S., Scholz, I., Matthiessen, J. K. F., Hess, W. R., and Voß, B. 2014, Comparative analysis of the primary transcriptome of *Synechocystis* sp. PCC 6803. *DNA Res.*, **21**, 527–39.
2. Gärtner, K., Klähn, S., Watanabe, S., et al. 2019, Cytosine N4-methylation via M.Ssp6803II is involved in the regulation of transcription, fine-tuning of DNA replication and DNA repair in the cyanobacterium *Synechocystis* sp. PCC 6803. *Front. Microbiol.*, **10**, 1–14.
